# Supplementary material for: Chromothripsis during telomere crisis is independent of NHEJ, and consistent with a replicative origin
Source: Genome Res. 2019 May;29(5):737–49. doi: 10.1101/gr.240705.118 (PMC6499312; doi:10.1101/gr.240705.118)
Supplement: Supplemental Material [file supp_gr.240705.118_Supplemental_file_1.zip › contigs/annotated_contigs/DB79/contig.2.DB79_length_493_mean_cov_5.47667342799.docx]

**DB79_length_493_mean_cov_5.47667342799**

```
TACAATTCTGGAGGCCAGAAGTCTGAAGTCAGTCACCCAGGGTGAAAATCAAGGCATTGGTAGGGCATATTTTTCTGGACGGTCTGGGG
 >chr7:116282248-116282634 - E=6e-218
AAGAATTTGTTTGTTTGACTTTTCTAGCATCTGGGGACTGAACCCTTTCTTGTATCACTCCAATCTTAGGCTTCCACTGTCACATCTAC

TTCTGTAGTCCAATGTCTCCCTACCTCTCTTATAAGAACACTTGTGATTAAACTTAAAGCTTACTTGGATAATCCAGGATAATCTCCTC

ATCTCAAAACCCTTGACTTAATCACACCTTCACAGTTCCCTTTGCCAAGTAAATGAACATTCACAGGTTCCAGGGATTAGGATGTGGAC

ATCTTGGGAAGGGGACATTATTCAGCTGA|C|TTGGGAGTCAGTTCATTATTGTGGAAGGGGGTTTGTTAGAGAATCTGTTCACCTCTT
                              >chr7:116427550-116427658 - E=2e-53
GCTCTCTCTTGCACTCTCTCACACCCATGTGATGCCTTCCACCATGTTAT
```
